# Evolution and Expression Divergence of the *CYP78A* Subfamily Genes in Soybean

**DOI:** 10.3390/genes9120611

**Published:** 2018-12-07

**Authors:** Ai-Hua Dai, Su-Xin Yang, Huang-Kai Zhou, Kuan-Qiang Tang, Guang Li, Jian-Tian Leng, Hui Yu, Yao-Hua Zhang, Jin-Shan Gao, Xia Yang, Yin-Jie Guo, Ning Jiang, Xian-Zhong Feng

**Affiliations:** 1 Key Laboratory of Soybean Molecular Design Breeding, Northeast Institute of Geography and Agroecology, Chinese Academy of Sciences, Changchun 130102, China; boxdah@126.com (A.-H.D.); yangsuxin@iga.ac.cn (S.-X.Y.); hkzhou@genedenovo.com (H.-K.Z.); tangkuanqiang@126.com (K.-Q.T.); liguang@iga.ac.cn (G.L.); lengjiantian@iga.ac.cn (J.-T.L.); yuhui@iga.ac.cn (H.Y.); zhangyaohua@iga.ac.cn (Y.-H.Z.); gaojinshan@iga.ac.cn (J.-S.G.); xyang@genetics.ac.cn (X.Y.); guoyinjie15@mails.ucas.ac.cn (Y.-J.G.); 2 University of Chinese Academy of Sciences, Beijing 100049, China; 3 Department of Horticulture, Michigan State University, East Lansing, MI 48824, USA; jiangn@msu.edu

**Keywords:** soybean, duplicate genes, evolution, *GmCYP78A*s, leaf size, seed size

## Abstract

Gene expression divergence is an important evolutionary driving force for the retention of duplicate genes. In this study, we identified three *CYP78A* subfamily genes in soybean, *GmCYP78A70*, *GmCYP78A57* and *GmCYP78A72*, which experienced different duplication events. *GmCYP78A70* was mainly expressed in leaf tissue and the vegetative phase, whereas *GmCYP78A57* was mainly expressed in floral tissue and seed, i.e., the reproductive phase. Expression of *GmCYP78A72* could be detected in all the tissues and phases mentioned above. The expression levels of *GmCYP78A70* and *GmCYP78A57* in different soybean cultivars showed positive correlations with leaf size and 100-seed weight, respectively. The population genetics analysis indicated that the three genes had experienced different selective pressures during domestication and improved breeding of soybean. Deciphering the function of this subfamily of genes may well prove useful to breeders for improving soybean’s agronomic traits.

## 1. Introduction

Whole-genome duplication (WGD) is an especially common event in plant lineages relative to animal lineages, with 50–70% or more of all angiosperms having undergone at least one detectable genome duplication event in their history [1,2]. Soybean experienced a Gamma whole-genome duplication before the origin of the rosids, ~130 to 240 million years ago (Mya); the legume WGD (Legume WGD, ~58 Mya), and the *Glycine* WGD in the *Glycine* lineage (*Glycine* WGD, ~13 Mya) [3,4,5,6]. This produced a highly duplicated soybean genome with nearly 75% of its genes present in multiple copies [6]. Genes that have duplicated copies should have more diversified expression profiles than single-copy genes during development [7]. In *Arabidopsis*, approximately 70% of duplicate pairs have significant differences in transcript levels [8]. Epstein [9] proposed that expression divergence is the first step in the functional divergence between duplicate genes and thereby increases the chance of retention of duplicate genes in a genome. It is now clear that gene duplication enables tissue or developmental specialization and increases expression diversity [10].

For duplicate genes, expression divergence is often used as a proxy indicator for the divergence of gene functions. The prevalence of expression sub-functionalization after polyploidization-variation in the relative expression of homologs among tissues in the polyploids has been assessed in several studies [11,12,13,14,15,16], and the differential expression between the duplicate genes has been shown to contribute to phenotypic variation [17]. In *Populus*, among the 60 class I PG (polygalacturonases) genes, 11 of them were specifically expressed in flowers. These 11 genes might acquire new functions related to flower development; thus, their evolutionary fates might be neo-functionalization and could contribute to the retention of these class I PG genes [18].

As the largest family of enzymatic synthesis genes by far, the *cytochrome P450*s (*CYP*s), which are excellent reporters of metabolism architecture and evolution [17,19], were positively influenced by the WGD in soybean. The *Arabidopsis* cytochrome P450 *KLUH* (*KLU*)/*CYP78A5* is expressed in leaf, flora and embryo tissues, and has been identified as a stimulator of plant organ growth [20,21]. In cultivated tomato, *SLKLUH*, encoding the ortholog of *KLUH*, shows a similar expression pattern and regulates plant architecture, ripening time and fruit mass [22]. Moreover, a single nucleotide polymorphism in the promoter of *SLKLUH* is highly associated with fruit mass [22]. The three closest homologs of *KLU* in soybean are *GmCYP78A70* (Glyma.01G061100), *GmCYP78A57* (Glyma.02G119600) and *GmCYP78A72* (Glyma.19G240800). *GmCYP78A72* has been proved to regulate the seed size and organ development [23]. Therefore, given the characteristics of the soybean genome and the function of *CYP78A* subfamily genes, soybean and its *GmCYP78As* forms an ideal subject for further studying the evolution and expression divergence of duplicated genes.

To that end, the present study analyzed the relationships between the origins of the three *GmCYP78A* genes and their relationships with the WGD in soybean. Moreover, clear expression divergence in different tissues was observed among the three genes. Our results showed that expression divergence largely explained the function variations of the *CYP78A* subfamily genes.

## 2. Materials and Methods

### 2.1. Gene Cloning

The *GmCYP78A70*, *GmCYP78A57* and *GmCYP78A72* gene fragments were amplified from the soybean cultivar Williams 82 (Supplementary Table S1). These PCR amplifications were carried out with KOD DNA polymerase in a 50 μL mix containing 200 μmol L^−1^ of dNTP, 0.4 μmol L^−1^ of each primer, 1 unit of KOD and 1 μL of cDNA. Reactions were performed at 95 °C for 2 min, and then cycled at 98 °C for 10 s, 55 °C for 15 s, 68 °C for 2 min for 35 cycles, and finally 68 °C for 5 min. The PCR products were cloned using a TA cloning vector (*pMD18-T*) and sequenced by Kumei Biology Co. (Changchun, Jilin, China). After verifying the sequences, these products were used for constructing an overexpression vector.

### 2.2. Phylogenetic Analysis and Gene Structure

We searched for the homologous genes of KLU/CYP78A5 from Phytozome (http://www.phytozome.net) for the following genomes: *Arabidopsis thaliana, Glycine max*, *Medicago truncatula*, *Phaseolus vulgaris* and *Physcomitrella patens*. Multiple sequence alignments of the amino acid sequences were performed in ClustalX (v 2.0.9) [24]. Unrooted phylogenetic and molecular evolutionary trees were constructed in MEGA7.0 (https://www.megasoftware.net/) using the neighbor-joining method [25]. The exon-intron organization was determined using the genome browser in RAP-DB (http://rapdb.dna.affrc.go.jp/). Gene structures were displayed using the Gene Structure Display Server (GSDS) (http://gsds.cbi.pku.edu.cn/chinese.php).

### 2.3. Ka/Ks Analysis and Calculating the Date of Duplication Events

The number of nonsynonymous substitutions per nonsynonymous site (Ka), and the number of synonymous substitutions per synonymous site (Ks), of the duplicated genes were calculated with the YN00 program in the PAML package [26]. The duplication date could be computed with the number of substitutions per silent site (Ks) [27]. A neutral evolutionary rate (λ) of substitutions per silent site per year was used to analyze the duplication history of the soybean genome. We calculated the duplication event dates using the equation T = Ks/2λ [28,29].

### 2.4. Constructs and Plant Transformation

The *35S::GmCYP78A*s vectors were constructed using a PCR-Restriction Enzyme ligation method. The coding sequences (CDS) were cloned into the *pMD18-T* TA cloning vector (Supplementary Table S1). The *ProGmCYP78A*s*::GUS* vectors were constructed by a PCR-based Gateway system. Specifically, ~3000-bp promoter sequences of *GmCYP78A*s were amplified (Supplementary Table S1), with a linker sequence at each end. The PCR products were recombined to the binary vector *pCAMBIA3301* (*35S* of the *GUS* had been digested with NcoI and HindIII) by using the In-Fusion enzyme (In-Fusion Cloning Kit, Clontech, Mountain View, CA, USA).

The Dongnong50 soybean cultivar served as control in all the experiments. The *pCYP78As::GUS* was mobilized to the *Agrobacterium tumefaciens* strain EHA105 and used for the transformation of soybean in an *Agrobacterium*-mediated transformation [30].

### 2.5. Gene Expression Analysis

Total RNAs were isolated from plant tissues with Trizol (TIANGEN Biotech, Beijing, China) following the manufacturer’s instructions. RNA quality was determined by a Nanophotomoter (Implen, München, Germany). The removal of genomic DNA residues, reverse transcription and cDNA synthesis were separately performed with 2 μg of total RNA and a FastQuant cDNA RT Kit (TIANGEN Biotech). Real-time PCR analysis of *GmCYP78A*s was performed with the FastStart Essential DNA Green Master (Roche, Shanghai, China) on a Stratagene Mx3005P (Agilent Technologies, Santa Clara, CA, USA, Supplementary Table S1). The relative expression levels were calculated from three replicates using the 2^−ΔΔ*C*t^ method after normalization to the *Actin11* control in soybean [31].

### 2.6. Measurement of Leaf Area

A digital camera (Nikon D90, Tokyo, Japan) was used to photograph the entire plants and their organ morphology. Leaf surface areas were measured from the obtained digital images of dissected organs in Image J.

### 2.7. In Situ Hybridization

Tissue fixation for *in situ* hybridization was carried out according to the protocol of Feng et al. [32]. The tissues were embedded in paraffin (Paraplast Plus, Sigma-Aldrich, Saint Louis, MO, USA) and sliced into 8 μm sections with a microtome (Leica, Wetzlar, Germany). The 3’-region of the *GmCYP78A*s cDNA was subcloned and used as a template to generate the sense and antisense RNA probes (Supplementary Table S1). Digoxigenin-labeled RNA probes were prepared with a DIG RNA Labeling Kit (T7/SP6) (Cat. no. 11175025910, Roche) according to the manufacturer’s instructions. The slides were observed under a bright field through a Leica microscope (DMI8, Leica).

### 2.8. Histology Analysis

For the GUS analysis, soybean tissues were incubated overnight at 37 °C in a 5-bromo-4-chloro-3-indolyl-b-glucuronic acid solution (Gold Biotechnology, Saint Louis, MO, USA). For clearing, tissues were treated with 70% ethyl alcohol. Light images from the GUS-stained tissues were obtained by an Olympus SZX7 (Olympus, Tokyo, Japan) stereomicroscope.

### 2.9. Artificial Selection Analysis

To analyze artificial selection, the selective sweep regions were detected with different parameters; namely, the nucleotide diversity (π) [33], and its π ratios (π_landrace_/π*_Glycine soja_*, π_improved cultivar_/π_landrace_), and the pairwise fixation index (F_st_) [34]. A 20-kb sliding window approach, with a 2-kb step-size, was applied to quantify these parameters using in-house PERL scripts. The top 1% and top 5% of outliers were selected to determine the region under evolutionary selection.

## 3. Results

### 3.1. Isolation and Structures of the GmCYP78A Subfamily Genes

**Figure 1:**
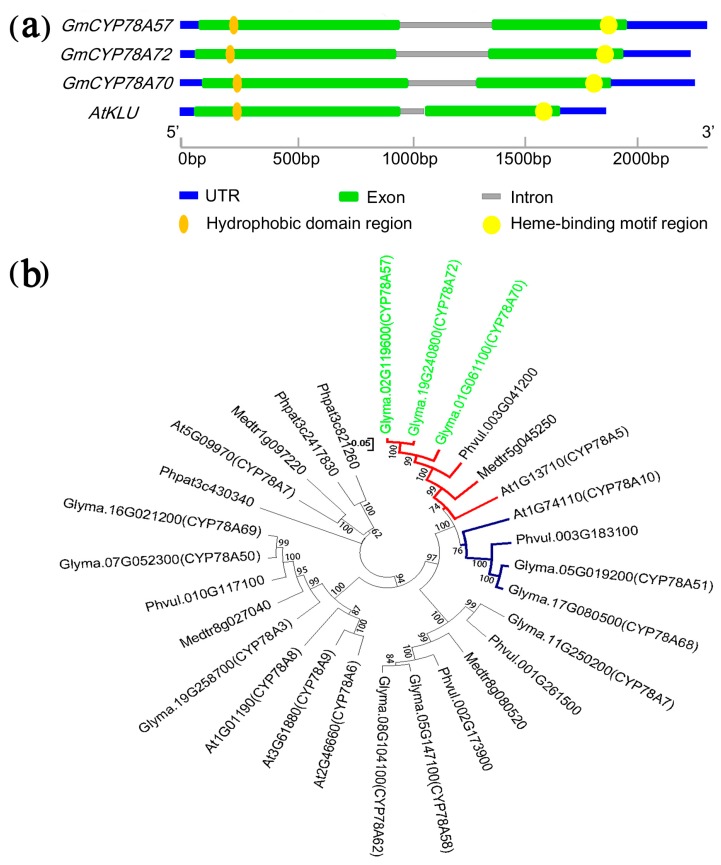
Gene structure and phylogenetic analysis of *GmCYP78A*s. (**a**) Schematic diagrams of the *GmCYP78A* gene structures. (**b**) Phylogenetic tree of GmCYP78As and other AtCYP78A5 homologous proteins from *Glycine max*, *Medicago truncatula*, *Phaseolus vulgaris*, *Physcomitrella patens* and *Arabidopsis*, constructed by MEGA7 using the neighbor-joining method. UTR: Untranslated Regions.

We isolated and cloned the genomic and CDS sequences of the three *GmCYP78A* subfamily genes (Supplementary Figure S1). All were consistent with the reference sequences in phytozome (http://www.phytozome.net) and they showed high identity (ranging from 63.30% to 64.20% at the amino acid level) to *Arabidopsis KLU*. The three genes showed similar sizes of the genomic DNA sequences and the CDS (Supplementary Table S2). Furthermore, we determined the intron-exon boundaries of the three genes. According to the annotation of the soybean genome, the three genes had very similar exon boundaries (Figure 1a); all three genes have two exons and one intron, and the exon lengths of the three genes varied by no more than 9 bp. This points to the conservation of the three *GmCYP78A* genes for coding capacity and the cis-elements for splicing.

The putative products of the *GmCYP78A* genes were all structurally similar to that of *KLU*, in that they had a conservative heme-binding motif in the C-terminal region and a hydrophobic helix structure in their N-terminal region (Figure 1a; Supplementary Figure S2) [35,36,37].

### 3.2. Evolution and Duplication of GmCYP78As

As we know, the WGD event that occurred 13 Mya did not occur in the progenitor shared with the genus *Phaseolus* or with the species *Medicago truncatula* [6,38,39]. So, according to the phylogenetic analysis of GmCYP78As and other CYP78A subfamily proteins from the five plant species (i.e., *G. max*, *M. truncatula*, *P. vulgaris*, *P. patens* and *Arabidopsis*), the *GmCYP78A*s loci (the red branch) occurring as duplicate pairs in soybean are each represented by a single locus in their counterparts in *P. vulgaris* and *M. truncatula* (Figure 1b). Co-expression analysis found that GmCYP78A57 and GmCYP78A72 are involved in the same expression network (Supplementary Figure S3). The two genes could regulate the cellular process, metal ion transport and metabolic process in the network, especially in the development of seed (Supplementary Table S3). However, GmCYP78A70 was not found in this network.

**Figure 2:**
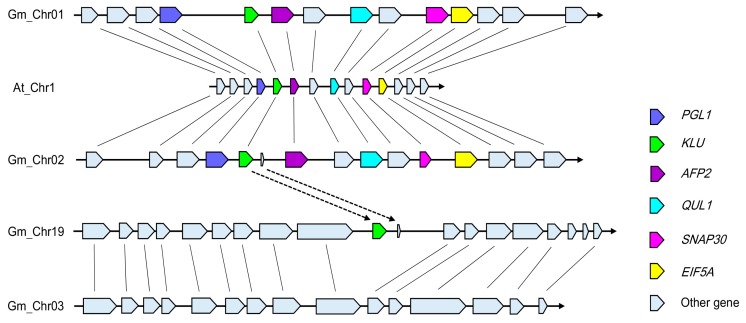
Synteny diagram plot of the soybean and *Arabidopsis* sequence assemblies surrounding the *Arabidopsis KLU* gene. The color key for specific genes is as follows: *PGL1* (6-phosphogluconolactonase 1, At1G13700)—blue; *KLU* (cytochrome P450, At1G13710)—green; *AFP2* (ABI five binding protein 2, At1G13740)—purple; *QUL1* (QUASIMODO2 LIKE 1, At1G13820)—magenta; *ANAP30* (Soluble N-ethylmaleimide-sensitive factor adaptor protein 30, At1G13860)—pink; and *EIF5A* (eukaryotic elongation factor 5A-1, At1G13890)—yellow. The syntenic matching genes in *G. max* are similarly colored. Solid black lines connect the homologous gene pairs.

To investigate the mechanism underpinning the emergence of three copies of *KLU/CYP78A5* in *G. max*, we performed a synteny analysis of the 132,521 bp region around *KLU—*starting at 4642346 bp, and ending at 4774867 bp on *Arabidopsis* chromosome1—using the webtool “MultiSyn” (http://202.31.147.159:62001/) in the *G. max* genome [40]. Interestingly, in the ensuing synteny plot, the genes and their arrangement were only highly consistent with those in chromosomes 01 and 02 of *G. max* (Figure 2; Supplementary Figure S4a). So, *GmCYP78A70* and *GmCYP78A57* were thus located in the syntenic blocks. By contrast, the region around *GmCYP78A72* on chromosome19 of *G. max* lacked a synteny relationship with the *KLU* locus in *A. thaliana*. Therefore, to determine the origin of *GmCYP78A72*, we used a 203,399 bp region around *GmCYP78A72—*starting at 48753501 bp, and ending at 48956900 bp on *G. max* chromosome 19—to search the soybean genome for homologous regions. The flanking sequence of *GmCYP78A72* showed strong synteny with a region on chromosome03 (Figure 2; Supplementary Figure S4b).

We also calculated the dates of the duplication events based on the non-synonymous nucleotide substitution rate (Ks) [41]. According to these results, the duplication event between *GmCYP78A70* and *GmCYP78A57* occurred 13.49 Mya, which coincided with the *Glycine* WGD in the *Glycine* lineage (Supplementary Table S4) [6], thus suggesting that this gene pair arose from the *Glycine* WGD event. However, the duplication event between *GmCYP78A57* and *GmCYP78A72* was estimated at ~3.87 Mya, much later than the *Glycine* WGD. Thus, it is likely *GmCYP78A72* was individually copied to its location after the duplication event between *GmCYP78A70* and *GmCYP78A57*.

### 3.3. Spatiotemporal Expression Patterns of GmCYP78A Homologs

**Figure 3:**
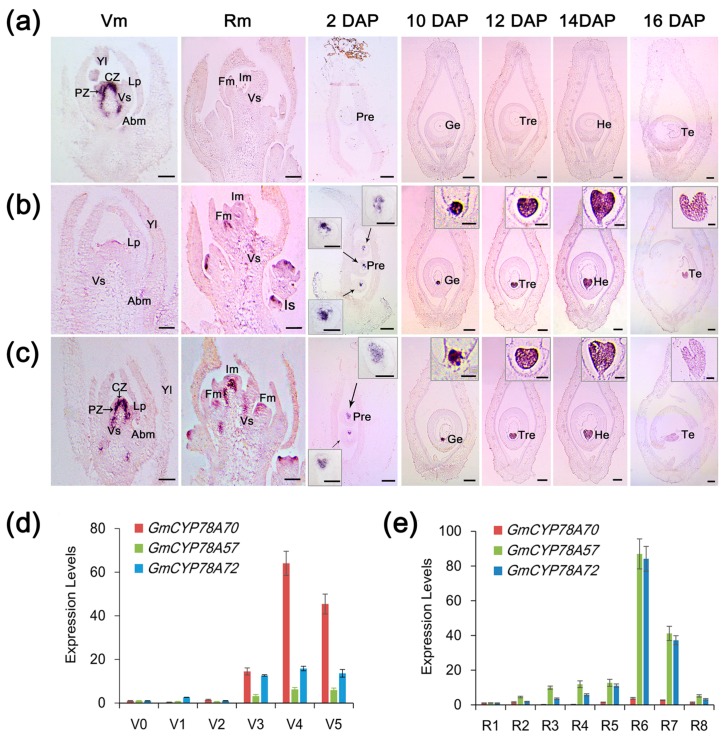
Spatiotemporal expression patterns of *GmCYP78A*s. (**a**–**c**) In situ hybridizations of *GmCYP78A70*, *GmCYP78A57* and *GmCYP78A72* were performed respectively in the vegetative meristem (Vm), reproductive meristem (Rm), and the carpel and young pod. Scale bars = 100 μm. For the boxed insets of (**b**,**c**) the scale bars = 20 μm. (**d**) Expression levels of *GmCYP78A*s at each stage of the vegetative phase (V0–V5). (**e**) Expression levels of the *GmCYP78A*s at each stage of the reproductive phase (R1–R8). Expression levels at the V0/R1 stage were set to 1, while those at other stages were adjusted accordingly. Data shown are mean ± SE (*n* = 3, biological replicates). CZ, central zone; PZ, peripheral zone; Abm, axillary bud meristem; Vs, developing vascular strand; Fm, floral meristem; Im, inflorescence meristem; Is, inflorescence stem; Pre, proembryo; Ge, global embryo; Tre, transition embryo; He, heart embryo; Te, torpedo embryo; DAP: days after pollination.

We examined the expression patterns of the three homologs via quantitative real-time PCR (qRT-PCR) using Williams82 (wild-type) mRNA and in situ hybridization. This analysis showed that the expression levels of the three *GmCYP78A* homologs were relatively low and stable from the vegetative 0 (V0) stage (when the cotyledons at node 0 are fully extended, but the unifoliate leaflets at node 1 have not yet unrolled) [42] to the V2 stage (when the first trifoliate leaflets at node 2 are fully expanded, but the second trifoliate leaflets at node 3 have not yet unfolded). However, expression increased dramatically in the V3 stage (when the second trifoliate leaflets are fully expanded but the third trifoliate leaflets remain rolled), especially for *GmCYP78A70* (Figure 3d). Next, we compared the expression patterns of the three genes at different reproductive growth stages. Interestingly, the expression of *GmCYP78A70* was down-regulated to a relatively low level whereas both *GmCYP78A72* and *GmCYP78A57* exhibited significantly higher levels of expression in stage R3 (pods are 5 mm in size at one of the four uppermost nodes on the main stem) through to R8 (95% of the pods have reached their full mature color), but especially in R6 (pod containing a green seed that fills the pod capacity at one of the four uppermost nodes on the main stem) (Figure 3e). The markedly high expression levels of *GmCYP78A57* and *GmCYP78A72* indicated their possible participation in the growth and development of soybean reproductive organs. Moreover, the shared expression pattern of *GmCYP78A57* and *GmCYP78A72* confirmed that they shared the same branch on the evolutionary tree (Figure 1b).

In the vegetative meristem (12 d), both the *GmCYP78A70* and *GmCYP78A72* transcripts were clearly detected in the central and peripheral zones of the shoot meristem and vascular strand (Figure 3a,c). *GmCYP78A70* was also expressed in the axillary bud meristem. However, only a very weak expression of *GmCYP78A57* was detected in these tissues (Figure 3b). In the reproductive meristem (35 d), *GmCYP78A57* and *GmCYP78A72* were highly expressed not only throughout the inflorescence and floral meristems, but also in the developing vascular strand (Figure 3b,c). Moreover, *GmCYP78A57* could be detected in the inflorescence stem. However, weak or no expression of *GmCYP78A70* occurred in the areas mentioned above (Figure 3a). In the young pod (10~16 DAP; days after pollination), abundant *GmCYP78A57* and *GmCYP78A72* transcripts were clearly observed in the embryo development of the globular, transition, heart and torpedo stages (Figure 2b,c), which together are responsible for cell proliferation toward the central vacuole. Moreover, *GmCYP78A57* showed higher expression levels than did *GmCYP78A72* during the process of embryo development, whereas their expression locations were similar. *GmCYP78A70* expression went undetected in the embryo. As the negative control, the sense probes did not provide any signal (Supplementary Figure S5). The localization of *GmCYP78A*s’ expression in both the meristem and the embryo was further corroborated by the real-time PCR results.

### 3.4. Activity and Transcription Factor Binding Sites of GmCYP78As Promoters Lead to Specialized Expression

**Figure 4:**
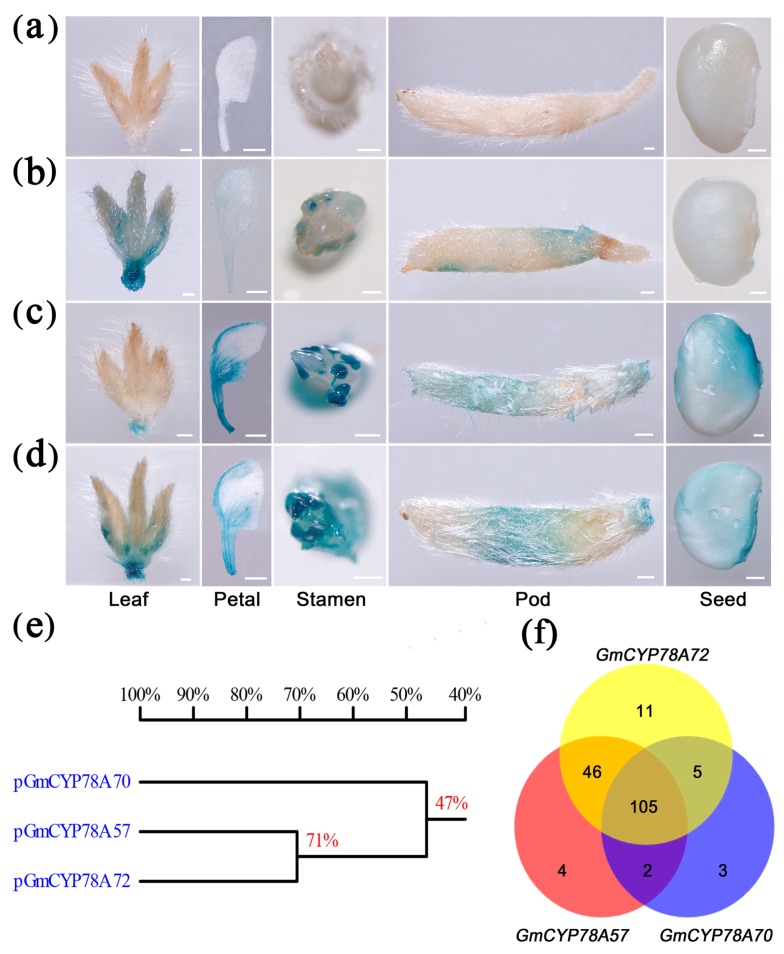
*GmCYP78A*s expression activities were monitored by *pGmCYP78As::GUS* transgenic expression. (**a**–**d**) Respective histochemical analyses of the control, *pGmCYP78A70::GUS*, *pGmCYP78A57::GUS* and *pGmCYP78A72::GUS* in developing trifoliate leaflets, petals, stamen, pod trichome and seeds. Scale bars = 1 mm in (a–d). (**e**) Comparison of the promoters of the *GmCYP78A* homologues of the gene. (**f**) Venn diagram of the transcription factors that could bind to the *GmCYP78A*s promoters.

The specific expression patterns may result from the different promoter activities of *GmCYP78A*s. Promoters of *GmCYP78A57* and *GmCYP78A57* showed higher identity than that of *GmCYP78A70* in the reference ‘Williams 82’ genome (Figure 4e). Therefore, we constructed the *ProCYP78A*s*::GUS* vectors (Supplementary Figure S6) and transformed them into the soybean cultivar Dongnong50. The tissue-specific expression patterns of the *GmCYP78A*s were examined by a histochemical assay of *GUS* activity. In the transformed plants of *ProCYP78A70::GUS*, the activity of GUS was high in young trifoliate leaflets (V2 stage) and pod trichome (R4 stage), whereas it was low or absent in the petal (R2 stage), stamen (R2 stage) and seed (R4 stage) (Figure 4b). Interestingly, *GmCYP78A57* promoter activity was concentrated in the petal, stamen, pod trichome and seed tissues, but not in the young trifoliate leaflets, for which only a relatively lower GUS activity was detected at the petiole’s end (Figure 4c). For the plants of *ProCYP78A72::GUS*, high GUS activity was detected in all the tissues and organs mentioned above (Figure 4d). These differences in promoter activities explained the expression patterns of *GmCYP78A*s, and thus confirmed our hypothesis.

Going further, we explored the transcription factor binding sites (TFBs) in the promoters of *GmCYP78A*s. The stable clusters of co-occurring TFBs coordinately regulate those gene sets associated with highly specific cellular activities [43,44]. Hence, we used the 2 kb promoter region of the *GmCYP78A*s and searched the *A. thaliana* transcription factor binding sites in the JASPAR database (http://jaspar.genereg.net/) (Figure 4f; Supplementary Table S5). Our results revealed 115 TFBs in the promoter region of *GmCYP78A70*, including three specific transcription factors: BZR1, SOD7 and DPA4. In *Arabidopsis*, overexpression of the mutant *bzr1-1D* gene increased cell elongation and led to a longer petiole and larger rosette leaves in the overexpression seedlings [45]. The CACTTG sequence is reportedly recognized by the transcription factors SOD7 and DPA4, and those two factors acted redundantly to regulate seed size by directly repressing *KLU* expression [46]; interestingly, since there is a CACTTG sequence only in the promoter of *GmCYP78A70*, this might help explain the specialized expression of the *GmCYP78A*s. We found 157 TFBs in the promoter region of *GmCYP78A57*, among which we identified four specific ones: ZAP1, F3A4, SMZ and TCP4. In *Arabidopsis*, the miR319a-targeting of *TCP4* was critical for petal growth and development [47]. An AP2-like transcription factor, SMZ, which repressed flowering and was a target of the regulatory miRNA172, functioned together with related proteins to directly regulate *FLOWERING LOCUS T* (*FT*) expression [48]. For *GmCYP78A72*, there were 167 TFBs and 11 specific ones—CRF4, PIF1, LFY, RAX3, ERF109, SPL7, TCP16, TCP5, FHY3, ABI5 and ABF3—in its promoter region, which had the most abundant gene-specific TFBs among the three soybean genes. In *Arabidopsis*, ERF109 mediated the cross-talk between jasmonic acid and auxin biosynthesis during the formation of lateral roots [49]. In addition, BRASSINOSTEROID INSENSITIVE2 (BIN2) could interact with ABSCISIC ACID INSENSITIVE5 (ABI5) to mediate the antagonism of brassinosteroids to abscisic acid during the phase of seed germination in *Arabidopsis* [50]. The ABA-responsive element binding factors ABF3 and ABF4 functioned in ABA signaling, and constitutive overexpression of either in *Arabidopsis* resulted in ABA hypersensitivity and other ABA-associated phenotypes, such that the transgenic plants had reduced transpiration and enhanced drought tolerance [51]. Since many of these transcription factors are expressed in the vegetative stages of plants, this likely explains why *GmCYP78A72* was not only present in the reproductive tissues, but in the vegetative ones as well.

### 3.5. Correlations of Expression Levels of GmCYP78As and Leaf Size in Soybean

**Figure 5:**
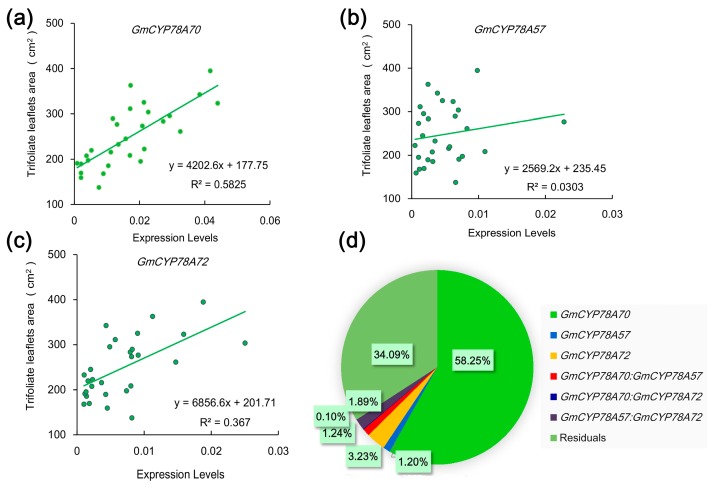
Relationships between the leaf area and the expression level of *GmCYP78A*s in leaves. (**a**–**c**) Linear regressions between the leaf areas and the expression levels of *GmCYP78A70*, *GmCYP78A57* and *GmCYP78A72*, respectively. Data shown are mean ± SE (*n* = 3). (**d**) The respective contribution of *GmCYP78A*s expression to leaf area (percentage of variation explained). For the statistical analysis of relationship between gene expression level and trait, a linear model was fitted with the R function lm. The model was fitted with the following terms: “trait ~ GmCYP78A70 + GmCYP78A57 + GmCYP78A72 + GmCYP78A70:GmCYP78A57 + GmCYP78A70:GmCYP78A72 + GmCYP78A57:GmCYP78A72”, where the individual effect of each gene and the interaction effects of gene pairs were included.

The specific expression patterns of the *GmCYP78A*s indicate the possibility of correlations between their expression levels and leaf size in the different soybean cultivars. To investigate this hypothesis, we tested the relationships between the leaf area and *GmCYP78A*s expression in 30 soybean cultivars at the V5 stage. The qRT-PCR analysis of leaf samples showed a significant positive correlation between the area of trifoliate leaflets and the expression levels of *GmCYP78A70* and *GmCYP78A72*. The Person’s *r* correlation coefficients of *GmCYP78A70* and *GmCYP78A72* were 0.76 (R^2^ = 0.5825) and 0.61 (R^2^ = 0.3670) (Figure 5a,c), respectively. However, *GmCYP78A57* did not show any significant correlation with leaf area (R^2^ = 0.0303) (Figure 5b). Moreover, we calculated the respective contribution of each gene’s expression level to the leaf size trait via regression analysis, finding that *GmCYP78A70* explained 58.25% of the variation in the enlargement of leaf size (Figure 5d), while the contributions of *GmCYP78A57* and *GmCYP78A72* were both relatively low.

We categorized the promoters of *GmCYP78A*s by genotype according to the sequences in 302 soybean accessions [52], and then amplified and sequenced the specific SNP (single nucleotide polymorphism) regions. We found that cultivars with “Genotype 1” and “Genotype 2” promoters showed higher expression levels of *GmCYP78A70*, and also produced trifoliate leaflets that were larger in area. “Genotype 3” consisted mostly of cultivars which showed lower expression levels of *GmCYP78A70* and smaller-sized trifoliate leaflets (Supplementary Table S6).

### 3.6. Correlations between the Expression Levels of GmCYP78As and the 100-Seed Weight in Soybean

**Figure 6:**
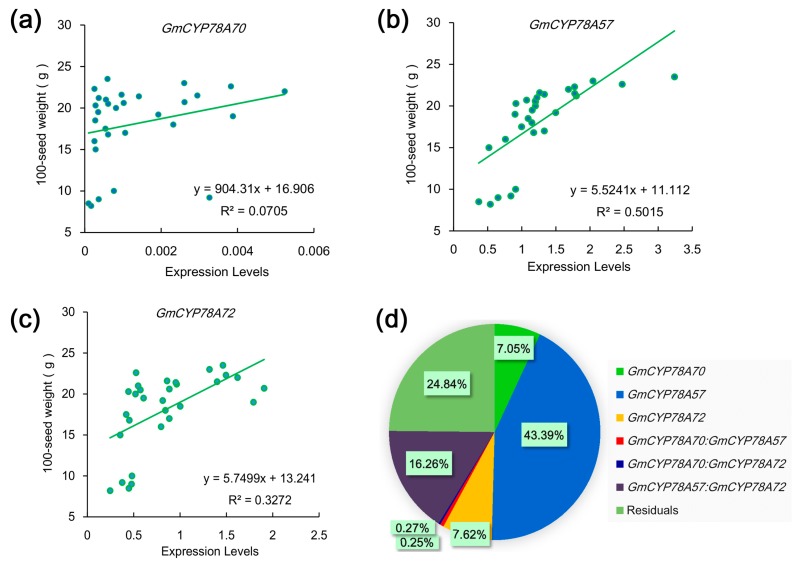
Relationships between the 100-seed weight and the expression level of *GmCYP78A*s in seeds. (**a**–**c**) Linear regressions between the 100-seed weights and the expression levels of *GmCYP78A70*, *GmCYP78A57* and *GmCYP78A72*, respectively. Data shown are mean ± SE (n = 3). (**d**) The respective contribution of *GmCYP78A*s expression to 100-seed weight (percentage of variation explained). For the statistical analysis of relationship between gene expression level and trait, a linear model was fitted with the R function lm. The model was fitted with the following terms: “trait ~ GmCYP78A70 + GmCYP78A57 + GmCYP78A72 + GmCYP78A70:GmCYP78A57 + GmCYP78A70:GmCYP78A72 + GmCYP78A57:GmCYP78A72”, where the individual effect of each gene and the interaction effects of gene pairs were included.

Larger seeds offer the newly germinating seedling a larger initial supply of nutrients which increases their competitiveness during seedling establishment and strengthens their tolerance to adverse environmental conditions [53]. Since *GmCYP78A72* is involved in regulating seed size, we speculated that those soybean cultivars with larger seeds might exhibit higher expression of *GmCYP78A72.* To test this hypothesis, we analyzed the correlation between the expression levels of *GmCYP78A*s and the 100-seed weight. We used 30 soybean cultivars on the basis of different 100-seed weights to test the expression levels of the three genes in seeds that were in the late maturation stage [54]. The 100-seed weight and the expression levels of *GmCYP78A57* and *GmCYP78A72* were positively correlated, with Person’s *r* coefficients of 0.71 (R^2^ = 0.5015) and 0.57 (R^2^ = 0.3272) (Figure 6b,c), respectively, whereas that of *GmCYP78A70* was weak, at 0.27 (R^2^ = 0.0705), which meant the *GmCYP78A70* expression level and the 100-seed weight were not related (Figure 6a). To further determine the contribution of *GmCYP78A*s towards seed size improvement, we fitted regression models between the 100-seed weight and the expression levels. The percentage contributions of *GmCYP78A57*, *GmCYP78A70* and *GmCYP78A72* were 43.39%, 7.05% and 7.62%, respectively (Figure 6d). Hence, *GmCYP78A57* significantly regulated the growth of seeds and its contribution was higher than that of *GmCYP78A70* and *GmCYP78A72* combined.

We genotyped two kinds of *GmCYP78A57* promoters in the 30 soybean cultivars, “Genotype 4” and “Genotype 5”. The expression levels of *GmCYP78A57* in the cultivars with “Genotype 4” were unremarkable when compared with those of “Genotype 5” (Supplementary Table S7). However, the lowest expression levels of *GmCYP78A57* were concentrated solely among those cultivars with “Genotype 5” promoters, and their corresponding 100-seed weights were also relatively small.

### 3.7. GmCYP78As Experienced Different Domestication and Improvement in Soybean

**Figure 7:**
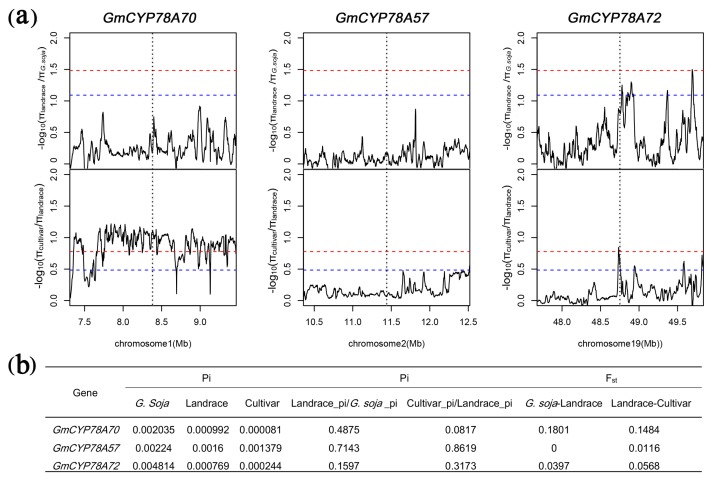
Selective pressure analysis for *GmCYP78A*s. (**a**) π ratios of the *GmCYP78A*s regions between different soybean populations. The red and blue dotted lines respectively represent the top 1% and top 5% thresholds selected to determine the region under evolutionary selection. The gray vertical lines correspond to the location of each *GmCYP78A*. (**b**) Index values relate to selective sweep in coding regions of *GmCYP78As*.

Expression levels of the *GmCYP78A*s showed remarkable positive correlations with leaf area and with seed size, both of which are important agronomic traits. As such, we examined the selective pressure by diversity (π) and the π ratio between populations and the genomic sequences of *GmCYP78A*s in 302 wild and cultivated accessions [52] (Figure 7; Supplementary Figure S7; Supplementary Table S8). The π ratio of the *GmCYP78A70* region was low between *Glycine soja* and landrace, whereas it was extremely high between landrace and the improved cultivars (Figure 7a). This result indicates that *GmCYP78A70* may have experienced weak selection during the domestication of soybean. but intense selection during its improvement. For the *GmCYP78A72* region, the π ratio between *G. soja* and landrace could reach the top 5% threshold range, whereas π ratio between landrace and the improved cultivar reached the top 1% threshold section (Figure 7a). Moreover, the π value of *GmCYP78A72* in *Glycine soja* was twice as high as those of the other two genes (Figure 7b), while the π value of *GmCYP78A57* was similar to that of *GmCYP78A70* (Figure 7b). However, all the π ratios of the *GmCYP78A57* region were very low (Figure 7a). Collectively, these results indicated that the *GmCYP78A70* region only experienced improvement breeding, while *GmCYP78A72* experienced both domestication and improvement breeding.

The Ka/Ks ratios of the three *GmCYP78A* genes demonstrated that they all evolved under purifying selection (Ka/Ks < 1) (Supplementary Table S6) [55]. One study suggested that sub-functionalized gene copies, when they show transcriptional divergence across either tissues or cell types, are expected to undergo purifying selection [56].

## 4. Discussion

Gene duplication plays a central role in plant diversification as a key process that forms the raw material necessary for adaptive evolution. Our study focused on the evolution and expression divergence of three *GmCYP78A* subfamily genes, which may be explained by the following hypothesis. First, the ancestor of the three *GmCYP78A*s was a single copy gene; it was expressed in both vegetative and reproductive organs. Then, *GmCYP78A70* and *GmCYP78A57* were derived from this single copy gene during the *Glycine* WGD process at ~13 Mya, followed by the formation of *GmCYP78A72* from *GmCYP78A57* on chromosome2. After the *Glycine* WGD, *GmCYP78A70* was only expressed in vegetative tissues whereas *GmCYP78A57* was only expressed in reproductive tissues. However, *GmCYP78A72* maintained the expression pattern of the ancestral gene. 

### 4.1. Contribution of Expression Divergence to the Retention of GmCYP78A Duplicate Genes

Previous studies have shown that divergence in gene expression contributes to the retention of duplicate genes [18,57]. In our work here, a clear divergence in the expression patterns was found among the *GmCYP78A* subfamily members. *GmCYP78A70* is expressed specifically in the leaf primordia, while *GmCYP78A57* is expressed mainly in floral organs and seeds; yet, *GmCYP78A72* is expressed in all the aforementioned tissues. Nevertheless, the expression levels of the three genes increased according to the growth of the regulated organs, suggesting that the expression divergence in these duplicated genes could be attributed to functional requirements. The *CYP78A* subfamily genes play important roles in various plant developmental processes, such as organ growth, anther and pollen grain maturation, and fruit development [20,22,58]. Angiosperms possess more complex organ systems and structures than do gymnosperms, and when these are newer they may require more CYP78As to maintain their biological functions. Interestingly, the three *GmCYP78A* genes showed significant expression in leaf, flower and seed. This is a powerful reason for the retention of the three duplicate genes in soybean’s evolutionary history.

### 4.2. GmCYP78A Subfamily Underwent Artificial Selection during Soybean Breeding

In soybean, *GmCYP78A10* is an orthologous gene of *Arabidopsis AtCYP78A10*, which is a subfamily member of the *KLU*. The allelic variation of *GmCYP78A10* is only associated with soybean seed size and pod number, but not with other domesticated traits [59]. In our study, however, we demonstrated that the expression levels of *GmCYP78A57* and *GmCYP78A72* were actually very high in the cultivars. Furthermore, a remarkable correlation was found between the leaf size and the expression levels of *GmCYP78A70* and *GmCYP78A72*. Moreover, the 100-seed weight of the soybean cultivars also showed a significant correlation with the expression levels of *GmCYP78A57* and *GmCYP78A72*. This suggests that after an extended period of artificial selection, the expression of all three genes has been enhanced, especially in those cultivars characterized by larger leaves and seeds.

Based on the population differentiation rate among the wild relatives, landraces and improved cultivars, *GmCYP78A72* has experienced continued selection during soybean domestication and modern breeding. Upon domestication, seed size was the most important trait sought after during artificial selection, and the variability in the *GmCYP78A72* region provided great potential and a direct target for selection since it controls seed size. Meanwhile, its expression correlation with leaf size represents an indirect target for artificial selection, in that a large leaf size is often correlated with a higher photosynthesis rate, which provides the ultimate resource for large seeds or high yields. Under these circumstances, then, it is not at all surprising that *GmCYP78A72* was the initial target for selection.

In the modern breeding era, particularly given the widespread application of chemical fertilizers, which enables extensive vegetative growth, further selection on leaf size might be more efficient to leverage the yield potential of soybean. In this case, breeders may wish to fully consider the photosynthesis efficiency of leaves; and if so, *GmCYP78A70* would experience stronger artificial selection in any improved cultivar breeding. Given the fact that the expression of *GmCYP78A57* is significantly correlated with seed size, while it has not supposed to experience significant artificial selection. However, this may indicate the potential of *GmCYP78A57* for improving seed size under future breeding selection scenarios. Hence, the cultivars featuring larger leaves and seeds, most of which showed a relatively high expression of the three genes, could be easily selected by breeders as the ‘chassis’ varieties in breeding new elite varieties of soybean. The relatively high expression levels of *GmCYP78A57* and *GmCYP78A72* in the larger soybean cultivars make them ideal candidates for the future breeding of large-seed soybean plants.

## Supplementary Materials

The following are available online at http://www.mdpi.com/2073-4425/9/12/611/s1, Figure S1: Isolation of the *GmCYP78A*s from soybean, Figure S2: Conserved sites in the CYP78As from soybean, *Arabidopsis*, rice, *Medicago truncatula*, *Vigna angularis* and *Phaseolus vulgaris*, Figure S3. Co-expression networks of GmCYP78A57 and GmCYP78A72, Figure S4: Synteny plots of soybean and Arabidopsis sequence assemblies surrounding the *AtKLU* and *GmCYP78A72* genes, Figure S5: Sense control of the in situ hybridization for *GmCYP78A*s, Figure S6: Diagrams illustrating the constructs used for transformation, Figure S7: Selective pressure analysis across the whole genome, Table S1: Primers used in this study, Table S2: Sizes of the genomic DNA sequences and the CDS, Table S3: Ontology analysis of the genes in the cluster, Table S4: Ka/Ks analysis and estimate of the absolute dates between the duplicated *GmCYP78A* genes, Table S5: Transcription factors that could bind to the *GmCYP78A*s promoters, Table S6: Genotyping of the *GmCYP78A70* promoters in 30 soybean cultivars, Table S7: Genotyping of the *GmCYP78A57* promoters in 30 soybean cultivars, Table S8: Threshold value of each index.
