# Supplementary material for: Evolution and Expression Divergence of the CYP78A Subfamily Genes in Soybean
Source: Genes (Basel). 2018 Dec 7;9(12):611. doi: 10.3390/genes9120611 (PMC6316016; doi:10.3390/genes9120611)
Supplement: Supplementary file 1 [file genes-09-00611-s001.pdf]

genes

MDPI

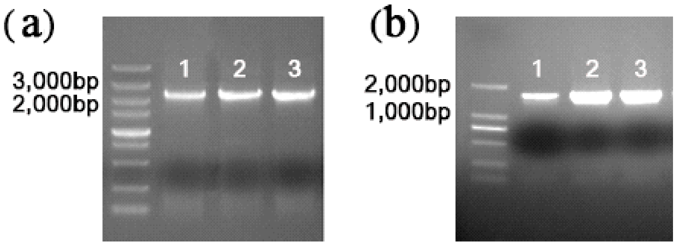

Figure S1. Isolation of the *GmCYP78As* from soybean. (**a**) PCR amplification of the genomic regions of *GmCYP78As* in wild-type Williams82. Lanes 1 to 3 represent *GmCYP78A70*, *GmCYP78A57* and *GmCYP78A72*, respectively, amplified by OL4100, OL0330, OL0388. (**b**) PCR-amplification of the CDS regions of *GmCYP78As* in wild-type Williams82 cDNA. Lanes 1 to 3 represent *GmCYP78A70*, *GmCYP78A57*, *GmCYP78A72*, respectively, and amplified by OL5684, OL5686 and OL5688.

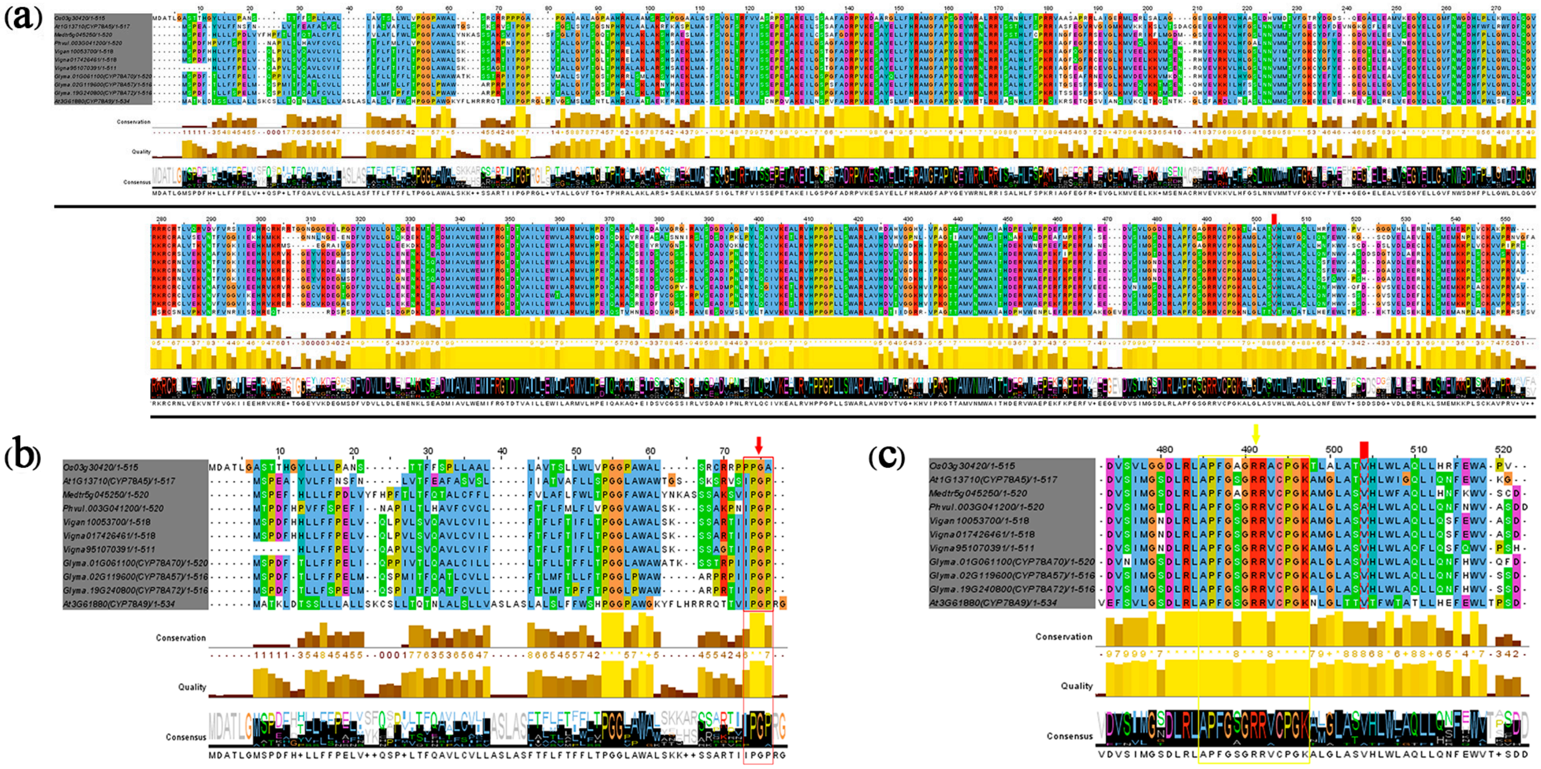

Figure S2. Conserved sites in the CYP78As from soybean, Arabidopsis, rice, *Medicago truncatula*, *Vigna angularis* and *Phaseolus vulgaris*. (**a**) Conserved sites in the CYP78As. (**b**) Hydrophobic helix structure in their N-terminal region (red arrow). (**c**) Heme-binding motif in their C-terminal region (purple arrow).

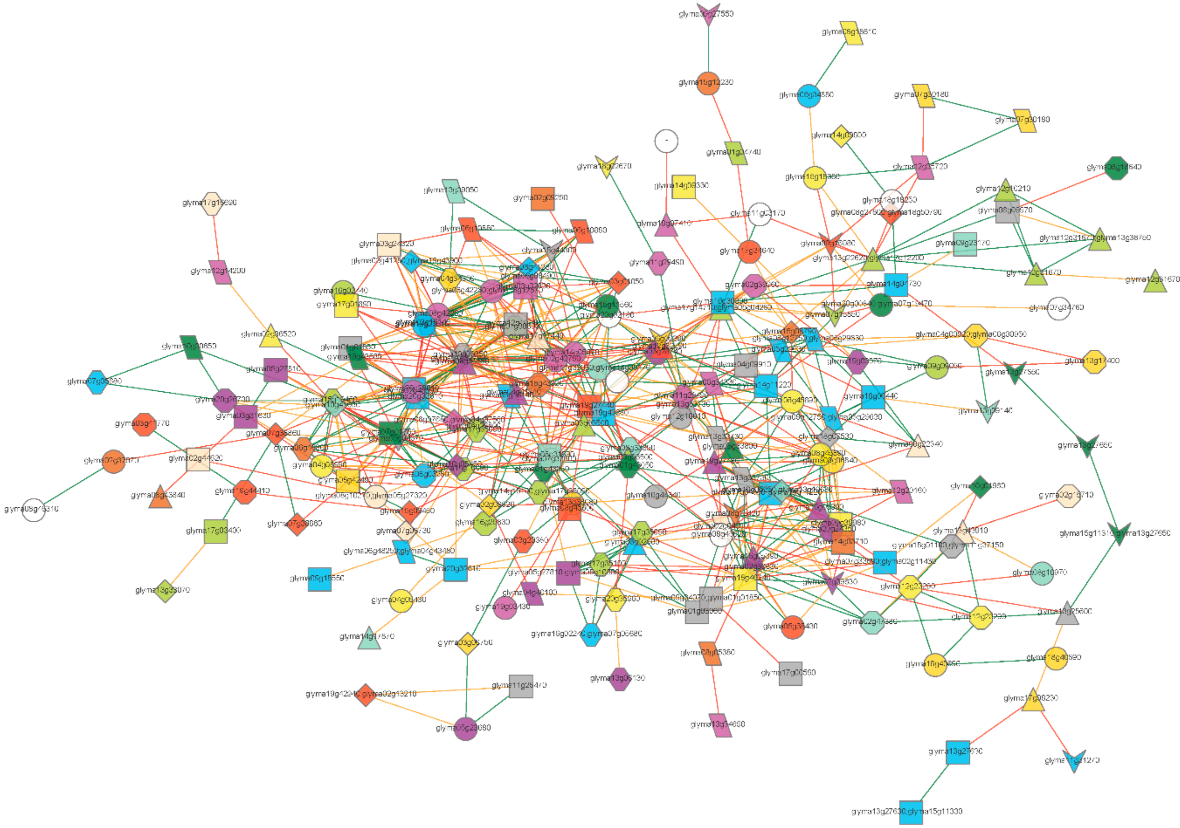

Figure S3. Co-expression networks of *GmCYP78A57* and *GmCYP78A72*.

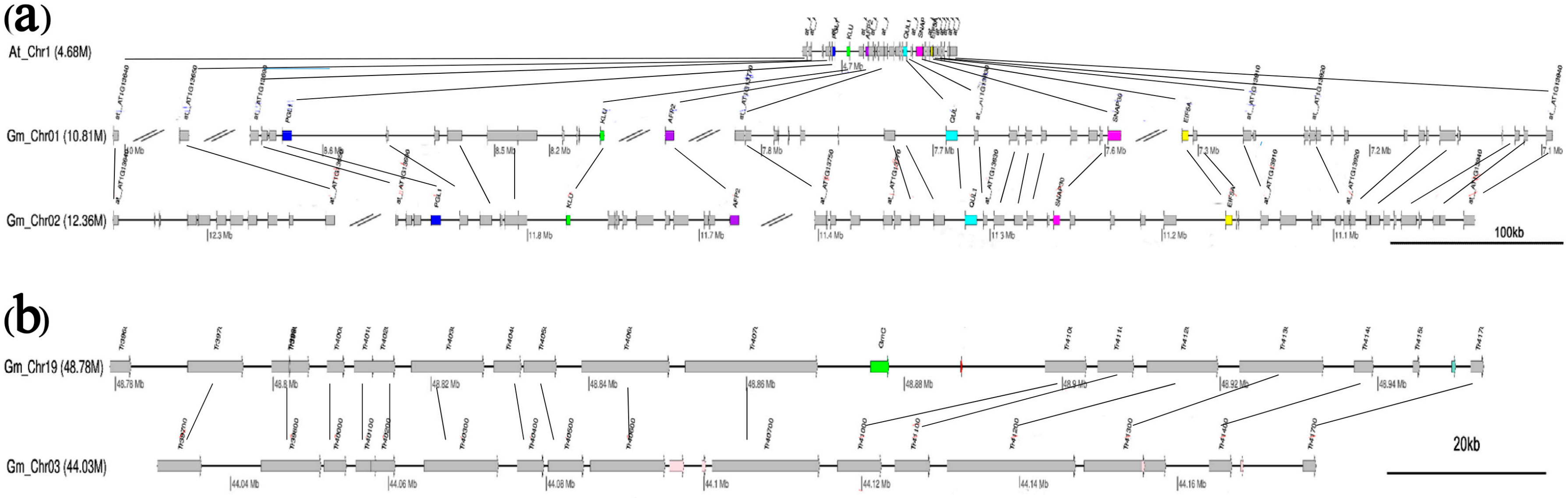

Figure S4. Synteny plots of soybean and Arabidopsis sequence assemblies surrounding the *AtKLU* and *GmCYP78A72* genes. (**a**) Synteny plot of the soybean and Arabidopsis sequence assemblies surrounding the *AtKLU*. (**b**) Synteny plot of the soybean sequence assemblies surrounding *GmCYP78A72*. The color key for specific genes is as follows: *PGL1* (6-phosphogluconolactonase 1, At1G13700) – blue; *KLU* (Cytochrome P450, At1G13710) – green; *AFP2* (ABI five binding protein 2, At1G13740) – purple; *QUL1* (QUASIMODO2 LIKE 1, At1G13820) – magenta; *ANAP30* (Soluble N-ethylmaleimide-sensitive factor adaptor protein 30, At1G13860) – pink; *EIF5A* (Eukaryotic elongation factor 5A-1, At1G13890) – yellow. The syntenic matching genes in *Glycine max* were also likewise colored. Change name: “gm__Glyma.19G2”to “Tr”.

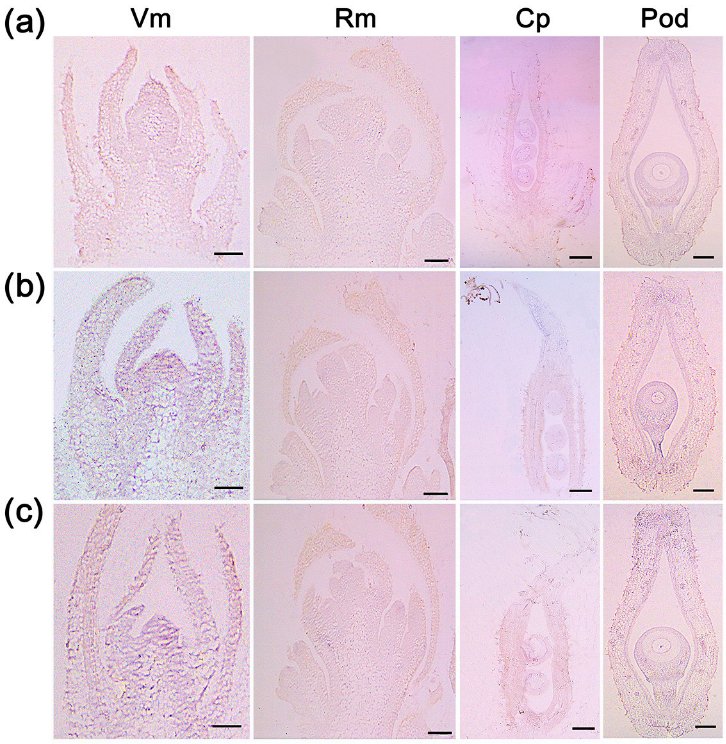

Figure S5. Sense control of the *in situ* hybridization for *GmCYP78As*. (**a-c**) Sense control of *GmCYP78A70*, *GmCYP78A57* and *GmCYP78A72* in vegetative meristem (Vm), reproductive meristem (Rm), and carpel (Cp) and young pod, respectively. Scale bars = 100 μm in (a-c).

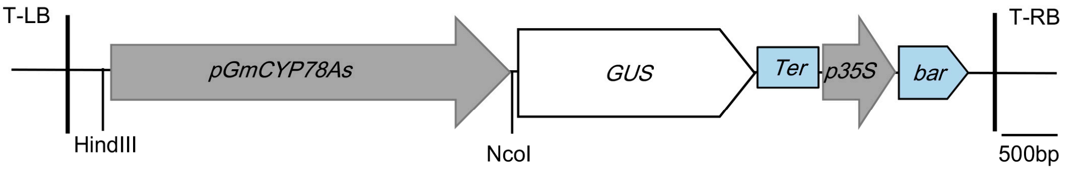

pGmCYP78As::GUS

Figure S6. Diagrams illustrating the constructs used for transformation. *pGmCYP78As::GUS*, contains the 3kb promoter regions of the *GmCYP78As* and *GUS* reporter.

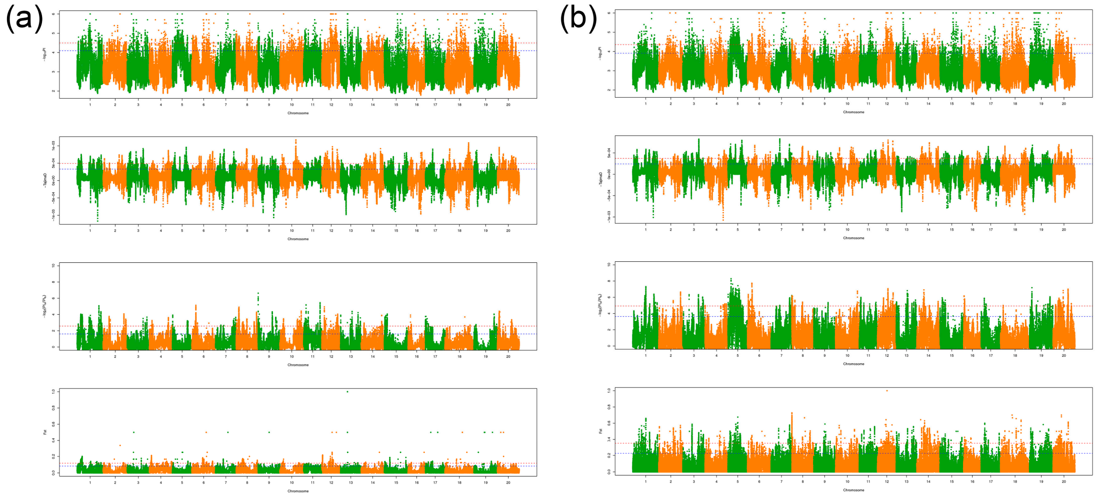

Figure S7. Selective pressure analysis across the whole genome. (**a**) Selective pressure between *Glycine soja* and landrace. (b) Selective pressure between landrace and the improved cultivar. Density of polymorphism (-log_{10}Pi), selection statistics (-Tajima's *D*), diversity between two populations (-$log_2 Pi_a/Pi_b$ and F_{st}), and genome annotation are shown (from top to bottom). Top 1% and 5% thresholds are shown in red and blue dotted lines, respectively.

Table S1. Primers used in this study.

Primers	Illustration	Locus	Forward sequence 5′→3′<br>Reverse sequence 5′→3′
OL4100	GmCYP78A70	Glyma.01G061100	AGTTCCAGCACAATAACCTCTTCATC<br>GCAACAGCAACCCTAGGCA
OL5684	GmCYP78A70<br>CDS	Glyma.01G061100	ATGTCACCAGATTTTACACTCTTGTT<br>GCAACAGCAACCCTAGGCA
OL0330	GmCYP78A57	Glyma.02G119600	GCACAACAAGCTCTTCATTTCTCC<br>ACGACCCACACAAAACAAAACCTC
OL5686	GmCYP78A57<br>CDS	Glyma.02G119600	ATGTCACCAGATTTCACACTTTTGT<br>GCAACAGAAACCCTAGGCACA
OL0388	GmCYP78A72	Glyma.19G240800	TTCAGCACACCGAATTCATCTCTCC<br>ACCACCTCCCGAATTCAACACACAT
OL5688	GmCYP78A72<br>CDS	Glyma.19G240800	ATGTCACCAGATTTTACACTCTTGTT<br>GCAAACAGAAACCCTAGGCAC
OL5209	Probe	Glyma.01G061100	TTAGATGACCTTTTTATTAAAATCTAATAC<br>CTATAACATCAAATATATAAAGGTAGGAAG
OL5211	Probe	Glyma.02G119600	GTTGTTCCCAAGGCTGTGCC<br>ATTAAACAACATAGAACGGCATGATC
OL5213	Probe	Glyma.19G240800	GTTCTTCGGTTGAGTTGGTTTG<br>TGCTGCAAATACACTTAATTAACAACT
OL5822	Promoter	Glyma.01G061100	TTGCATCACTTGTTCACCACACC<br>TTTTTGTTTTGTTTTGGTTTTTTTGTG
OL5824	Promoter	Glyma.02G119600	TGAGCTGAGGGTGAGATCACTGTT<br>TTTTGGTGAAAGAAAAGAAAGTGTGG
OL5826	Promoter	Glyma.19G240800	TTCATTAAACATCAGCGGTAATGTT<br>TTTTGGTAGTGTTGTTGCAAAGAA
OL2187	q RT-PCR	Glyma.01G061100	GAAGTTAAGAAGGTTATGAAGGATAACCG<br>CACCCTCACCCTCGTAGAACTCAT
OL2816	q RT-PCR	Glyma.02G119600	TCTCGTGGGCTCGCCTTGCT<br>CCGCTCGGGCCTAAACTTCT
OL1009	q RT-PCR	Glyma.19G240800	GATGTTTTGCTTGATTTGGAGAAGG<br>CTTTGCTTGGATTTCAGGGTGGAG

Table S2. Sizes of the genomic DNA sequences and the CDS.

Gene	Size of the genomic DNA sequence (bp)	Size of the CDS (bp)
GmCYP78A70	2341	1563
GmCYP78A57	2425	1551
GmCYP78A72	2350	1551

Table S3. Ontology analysis of the genes in the cluster.

ID	Domain	Description	Terms in network	Terms in background	Term enrichment p-value
GO:0050794	BP	regulation of cellular process	15	2794	0.0
GO:0048522	BP	positive regulation of cellular process	2	222	0.01
GO:0019222	BP	regulation of metabolic process	15	2120	0.0
GO:0060255	BP	regulation of macromolecule metabolic process	14	1834	0.0
GO:0043086	BP	negative regulation of catalytic activity	2	198	0.01
GO:0080090	BP	regulation of primary metabolic process	14	1881	0.0
GO:0006355	BP	regulation of transcription, DNA-templated	13	1537	0.0
GO:0006810	BP	transport	12	1912	0.0
GO:0044765	BP	single-organism transport	11	1332	0.0
GO:0071702	BP	organic substance transport	4	746	0.0
GO:0030001	BP	metal ion transport	2	277	0.01
GO:0055085	BP	transmembrane transport	6	690	0.0

Table S4. Ka/Ks analysis and estimate of the absolute dates between the duplicated *GmCYP78A* genes

Gene pair	Ka	Ks	Ka/Ks	Date (million years)	Purifying selection
GmCYP78A70/GmCYP78A57	0.0639	0.405	0.1579	13.49	Yes
GmCYP78A70/GmCYP78A72	0.0647	0.418	0.1549	13.92	Yes
GmCYP78A57/GmCYP78A72	0.0174	0.116	0.1496	3.87	Yes

Table S5. Transcription factors that could bind to the *GmCYP78A*s promoters.

Promoters	Transcription factors									
	ABI3	ARR18	BZR1	DOF5.6	ERF094	ERF7	MYB3	PIF3	TGA1	WRKY23	WRKY63
	AGL3	ATHB-12	CDC5	DOF5.7	ERF096	ERF8	MYB4	PIF4	TGA2	WRKY25	WRKY75
	AHL12	ATHB-6	CDF2	DRE1C	ERF098	FUS3	MYB46	PIF5	TGA5	WRKY30	WRKY8
	AIB	BEE2	CDF3	DREB1A	ERF105	GATA12	MYB55	RAP2-10	TGA7	WRKY38	SOD7
	ARF1	BHLH13	CMTA2	DREB1E	ERF11	GATA15	MYB59	RAP2-3	TRB2	WRKY40	DPA4
GmCYP78A70	ARF3	BHLH3	CMTA3	DREB2C	ERF112	GATA8	MYC2	RAV1	UNE10	WRKY43	
	ARF5	BHLH34	CRF2	ERF008	ERF13	HBI1	MYC3	3-Sep	WRKY12	WRKY45	
	ARF8	BIM1	DOF1.8	ERF018	ERF1B	KAN1	MYC4	SPT	WRKY15	WRKY48	
	ARR1	BIM2	DOF2.4	ERF039	ERF3	LEC2	NAC025	TCP19	WRKY18	WRKY57	
	ARR10	BIM3	DOF2.5	ERF043	ERF4	MYB111	NAC058	TCP2	WRKY2	WRKY60	
	ARR14	bZIP68	DOF5.3	ERF069	ERF6	MYB24	NAC92	TCP20	WRKY21	WRKY62	
	ABF2	ARF3	ATHB-6	CDF3	ERF008	ERF7	MYB24	NAC083	SPL12	WRKY12	WRKY60
	ABI3	ARF5	ATHB-7	CMTA2	ERF018	GATA11	MYB3	NAC92	T11I18.17	WRKY15	WRKY62
	AG	ARF8	BEE2	CMTA3	ERF039	GATA12	MYB4	NTL9	TCP15	WRKY18	WRKY63
	AGL1	ARR1	BHLH13	DOF1.8	ERF043	GATA15	MYB46	PI	TCP19	WRKY2	WRKY75
	AGL15	ARR10	BHLH3	DOF2.4	ERF069	GATA8	MYB55	PIF3	TCP2	WRKY21	WRKY8
	AGL27	ARR11	BHLH34	DOF2.5	ERF094	GATA9	MYB59	PIF4	TCP20	WRKY23	ZAP1
GmCYP78A57	AGL3	ARR14	BIM1	DOF5.3	ERF096	GT-1	MYC2	PIF5	TCP23	WRKY25	
	AHL12	ARR18	BIM2	DOF5.6	ERF098	HAT1	MYC3	RAP2-3	TCP4	WRKY30	
	AHL20	ARR2	BIM3	DOF5.7	ERF105	HAT5	MYC4	RAV1	TGA1	WRKY38	
	AHL25	ATHB-12	BZIP60	DRE1C	ERF11	HBI1	NAC025	RAV1(var.2)	TGA2	WRKY40	
	AIB	ATHB-15	bZIP68	DREB1A	ERF112	KAN1	NAC043	1-Sep	TGA5	WRKY43	
	AP1	ATHB-16	BZR2	DREB1E	ERF13	KAN4	NAC055	3-Sep	TGA7	WRKY45	
	AP3	ATHB-5	CCA1	DREB2C	ERF1B	LEC2	NAC058	SMZ	TRB2	WRKY48	
	ARF1	ATHB-51	CDF2	EDT1	ERF3	MYB111	NAC080	SOC1	UNE10	WRKY57	
GmCYP78A72	ABF2	ARF3	BEE2	CRF2	ERF043	FHY3	MYB111	NAC92	SPL3	TGA2	WRKY48
	ABF3	ARF5	BHLH13	CRF4	ERF069	FLC	MYB24	NTL9	SPL4	TGA5	WRKY57

	ABI3	ARF8	BHLH3	DOF1.8	ERF094	FUS3	MYB3	PI	SPL5	TGA7	WRKY60
	ABI5	ARR1	BHLH34	DOF2.4	ERF096	GATA10	MYB4	PIF1	SPL7	TRB2	WRKY62
	AG	ARR10	BIM1	DOF2.5	ERF098	GATA11	MYB46	PIF3	SPL8	WRKY12	WRKY63
	AGL1	ARR11	BIM2	DOF5.3	ERF105	GATA12	MYB55	PIF4	SPT	WRKY15	WRKY75
	AGL15	ARR14	BIM3	DOF5.6	ERF109	GATA15	MYB59	PIF5	SVP	WRKY18	WRKY8
	AGL27	ARR18	BZIP60	DOF5.7	ERF11	GATA8	MYC2	RAP2-10	T11I18.17	WRKY2	
	AGL3	ARR2	bZIP68	DRE1C	ERF112	GATA9	MYC3	RAP2-3	TCP15	WRKY21	
	AHL12	ATHB-12	BZR2	DREB1A	ERF13	GT-1	MYC4	RAV1	TCP16	WRKY23	
	AHL20	ATHB-15	CCA1	DREB1E	ERF1B	HAT1	NAC025	RAV1(var.2)	TCP19	WRKY25	
	AHL25	ATHB-16	CDC5	DREB2C	ERF3	HAT5	NAC043	RAX3	TCP2	WRKY30	
	AIB	ATHB-5	CDF2	EDT1	ERF4	KAN1	NAC055	1-Sep	TCP20	WRKY38	
	AP1	ATHB-51	CDF3	ERF008	ERF6	KAN4	NAC058	3-Sep	TCP23	WRKY40	
	AP3	ATHB-6	CMTA2	ERF018	ERF7	LEC2	NAC080	SOC1	TCP5	WRKY43	
	ARF1	ATHB-7	CMTA3	ERF039	ERF8	LFY	NAC083	SPL12	TGA1	WRKY45	

Table S6. Genotyping of the *GmCYP78A70* promoters in 30 soybean cultivars.

	Genotype	Cultivar	Expression level	Trifoliate leaflets area (cm^2)
1	ATGAATGGTACCGAATAC	Zhongzuo9228	0.017148018	311.238
		Heinong21	0.021345746	325.245
		Jinong12	0.029241102	295.355
		Bainong3	0.032509454	261.359
		Jinong13	0.038445355	342.517
		Jiunong29	0.041684412	394.739
2	GCGGATGGTACCGAATGC	Changnong8	0.020873849	273.453
		Longxuan09-502	0.017274163	362.752
		Changnong10	0.022676184	303.656
3	GTAGGCATCACTAAACGT	Jiunong22	0.007552905	137.536
		Hefeng25	0.002059006	159.186
		Heinong51	0.008924049	167.962
		Dongnong52	0.002059006	169.578
		Jilinxiaoli4	0.010434132	185.569
		Lingyin2	0.00208007	189.631
		Jiunong28	0.000890042	191.009
		Changnong10	0.020333357	195.265
		Sinong2	0.004232378	197.567
		Jiyu65	0.003738433	207.638
		Dongnong37	0.017060642	208.482
		Dongnong35	0.011216663	215.768
		Changnong14	0.005268748	219.586
		Beidou5	0.021472223	222.563
		Jilin44	0.013520427	232.892
		Heinong17	0.0157792	245.079
		Heinong4	0.013101578	276.752
		Chengdou6	0.027262392	283.593
		Changnong15	0.011813079	289.563
		Jinong11	0.043971908	323.162

Table S7. Genotyping of the *GmCYP78A57* promoters in 30 soybean cultivars.

	Genotype	Cultivar	Expression level	100-seed weight (g)
4	AAAGACAGCGTACAGTG GGTGGCCCCCGCGA	Jiyu89	1.174439381	16.8
		Henong59	0.996568408	17.5
		Jidadou1	1.497036067	19.2
		Heinong51	1.199157372	20
		Jiyu47	0.91342184	20.3
		Hefeng25	1.221759058	21
		Dongnong52	1.799513526	21.2
		Jiyu63	2.043627024	23
		Jidadou2	3.239217335	23.5
5	AAAGACAGCGTACAGCG GGTGGCCCCCGCGA	Jilinxiaoli4	0.538626422	8.2
		Dongnong50	0.368076675	8.5
		Jilinxiaoli6	0.651164001	9
		Suixiaolidou	0.83919776	9.2
		Tongnong14	0.90859146	10
		Lingyin1	0.518188249	15
		Lingyin2	0.75959113	16
		Jinong18	1.329762393	17
		Suinong30	1.147259741	18
		Dengke1	1.092967721	18.5
		Beidou5	0.900924298	19
		Dongsheng10	1.152216081	19.5
		Heihe36	1.200666068	20.5
		Jiyu62	1.19760371	20.6
		Jiunong22	1.070145108	20.7
		Jidadou3	1.331260349	21.4
		Jinong41	1.775196484	21.5
		Jiyu65	1.261142771	21.6
		Huajiang2	1.683326531	22
		Jiyu72	1.773246116	22.3
		Jilin40	2.471919113	22.6

Table S8. Threshold value of each index.

Threshold value	Pi			Pi		Fst	
	G. soja	Landrace	Cultivar	Landrace/G. soja	Cultivar /Landrace	G. soja-Landrace	Landrace-Cultivar
1%	0.000117	0.000044	0.000032	0.0329	0.1667	0.3487	0.1189
5%	0.000568	0.000122	0.000081	0.081	0.3274	0.2247	0.0833

© 2018 by the authors. Licensee MDPI, Basel, Switzerland. This article is an open access article distributed under the terms and conditions of the Creative Commons Attribution (CC BY) license (http://creativecommons.org/licenses/by/4.0/).
